# Dose–response relationships of psilocybin-induced subjective experiences in humans

**DOI:** 10.1177/0269881121992676

**Published:** 2021-03-04

**Authors:** Tim Hirschfeld, Timo T Schmidt

**Affiliations:** 1 Psychotropic Substances Research Group, Charité Universitätsmedizin, Berlin, Germany; 2 Department of Education and Psychology, Freie Universität Berlin, Berlin, Germany

**Keywords:** Phenomenology, psilocybin, dose–response relationship, subjective experience, meta-regression

## Abstract

**Background:**

Psilocybin is the psychoactive component in *Psilocybe* mushrooms (‘magic mushrooms’). Whether and how the quality of the psilocybin-induced experience might mediate beneficial health outcomes is currently under investigation, for example, in therapeutic applications. However, to date, no meta-analysis has investigated the dose-dependency of subjective experiences across available studies.

**Aim:**

Establishing dose–response relationships of the subjective experiences induced by psilocybin in healthy study participants and a comparison of patient groups.

**Method:**

We applied a linear meta-regression approach, based on the robust variance estimation framework, to obtain linear dose–response relationship estimates on questionnaire ratings after oral psilocybin administration. Data were obtained from the Altered States Database, which contains data extracted from MEDLINE-listed journal articles that used standardized and validated questionnaires: the Altered States of Consciousness Rating Scale, the Mystical Experience Questionnaire and the Hallucinogen Rating Scale.

**Results:**

Psilocybin dose positively correlated with ratings on most factors and scales, mainly those referring to perceptual alterations and positively experienced ego dissolution. Measures referring to challenging experiences exhibited small effects and were barely modulated by dose.

**Conclusion:**

Psilocybin intensified almost all characteristics of altered states of consciousness assessed with the given questionnaires. Because subjective experiences are not only determined by dose, but also by individual and environmental factors, the results may only apply to controlled laboratory experiments and not to recreational use. This paper may serve as a general literature citation for the use of psilocybin in experimental and clinical research, to compare expected and observed subjective experiences.

## Introduction

Psilocybin has been used for centuries by native cultures and more recently in modern research for its psychoactive properties (Schultes and Hofmann, 1979). It is a naturally occurring tryptamine (4-phosphoryloxy-N,N-dimethyltryptamine) and the primary psychoactive component in *Psilocybe* mushrooms (Hofmann et al., 1958; Tylš et al., 2014). After ingestion, psilocybin is rapidly dephosphorylated to psilocin (4-hydroxy-N,N-dimethyltryptamine), which is mainly responsible for the psychedelic effects via partial agonist action at serotonin type 2A (5-HT2A) receptors (Vollenweider et al., 1998), similar to other classic psychedelics (Nichols, 2016).

The potential beneficial and therapeutic effects of psilocybin are currently under investigation even though it is still unclear to what extent such effects are due to neurobiological mechanisms or due to the psychological experience of an altered state of consciousness (ASC) (Vollenweider and Preller, 2020). Psilocybin can induce profound ASC, comprising personally meaningful and spiritually significant mystical-type experiences (Griffiths et al., 2006, 2008, 2011; Pahnke, 1963). The quality of the experience seems to be associated with positive changes in mood, attitude and behaviour in healthy individuals (Griffiths et al., 2006, 2008, 2011, 2018; MacLean et al., 2011; Madsen et al., 2020; Studerus et al., 2011), and therapeutic outcomes in patients with alcohol use disorder (Bogenschutz et al., 2015), nicotine use disorder (Garcia-Romeu et al., 2015; Johnson et al., 2014, 2017), major depression (Carhart-Harris et al., 2016, 2018; Davis et al., 2020; Roseman et al., 2018) and cancer-related psychiatric distress (Agin-Liebes et al., 2020; Griffiths et al., 2016; Grob et al., 2011; Ross et al., 2016). While it is of particular interest for future investigations to know how psilocybin dose relates to the phenomenological quality of the ASC, no dose–response meta-analysis has been reported with regards to psychoactive properties. Such an analysis will also help to determine whether the acute psilocybin-induced experiences differ in specific patient groups.

Establishing dose–response relationships of subjective psilocybin experiences comes with several challenges. First, to establish dose–response relationships, the response measure must be accurately acquired across participants, which is challenging for psychological effects that depend on introspection (Cardeña et al., 2000). The gold standard for measuring ASC experiences is retrospective assessment with standardized and validated questionnaires (Cardeña et al., 2000; Passie, 2007; Schmidt and Majic, 2016). To date, multiple questionnaires have been developed to quantify different aspects of ASC phenomena. Such psychometric measures allow direct comparisons between induction methods, individuals’ responses, averaged group responses, and different experimental settings. Moreover, conducting laboratory studies with psilocybin is work- and cost-intensive as pharmacological intervention studies must conform to strong security standards to ensure the safety of participants (Nutt et al., 2013). Therefore, the amount of studies with controlled doses of psilocybin is limited. In addition, some studies report statistically dependent effect sizes by assessing the same sample of study participants multiple times, which constitutes a challenge for conventional meta-analytical approaches. A recently presented meta-analytical approach, the robust variance estimation (RVE) framework (Hedges et al., 2010; Tipton, 2015), permits the inclusion of small samples and statistically dependent effect sizes to determine reliable dose–response estimates. Dose–response relationships are usually best described by a sigmoid function, having a characteristic S-shaped curve that starts from no measurable response and converges at a maximum response that does not proportionally increase with dose. As the reported doses of psilocybin do not cover the upper and lower bounds of a sigmoid model, a linear function appears to be a suitable approximation for the dynamic range of the sigmoid dose–response function. Previous work also suggests a linear relationship of subjective experiences for medium to high doses of psilocybin is a good fitting model (Vollenweider and Kometer, 2010; Studerus et al., 2011). Taken together, the RVE meta-regression approach appears well-suited to determine linear dose–response relationships for psilocybin with the currently available data.

As it is well-known that the quality of the psilocybin-induced experience is not only determined by dose, but also by non-pharmacological factors like the psychological state of the individual and the setting of psilocybin administration (Hartogsohn, 2017; Leary et al., 1963; Zinberg, 1984), it is worth comparing dose–responses across studies to elucidate the variability of subjective experiences as well as the influence of non-pharmacological factors on response measures for dose-determination in future studies.

In this paper, we obtain estimates for dose–response relationships of the subjective experiences for orally administered psilocybin in healthy study participants in a controlled setting based on the data from the Altered States Database (ASDB; Schmidt and Berkemeyer, 2018), a collection of the currently available psychometric data on ASC experiences. In an additional analysis, we include data from patient populations to explore potential differences in their psilocybin-induced subjective experiences. Results of this analysis may be used for dose-determination in experimental and clinical studies.

## Methods

### Included data

We used the ASDB to identify peer-reviewed articles that contain suitable data for our meta-analysis. The data were obtained from the Open Science Framework repository in version ASDB_v1.1a_2020 (published in December 2020) (Schmidt, 2017). For the meta-analysis, we only included datasets in which the effects of orally administered psilocybin were investigated in healthy study participants and any of the questionnaires listed in Table 1 were applied. We therefore excluded the following studies from the main analysis: Grob et al. (2011), Bogenschutz et al. (2015) and Griffiths et al. (2016) because these studies investigated the effects of psilocybin in patient populations; Griffiths et al. (2018) because psilocybin administration was combined with meditation, and Carhart-Harris et al. (2011) because psilocybin was administered intravenously. Two articles, Wittmann et al. (2007) and Schmidt et al. (2012), reported the identical data as they were previously reported in another article and were therefore excluded from the analysis. Further, we found two articles that each reported data on a subsample of participants of a different study. We therefore included the data on the larger samples and excluded the data from Carter et al. (2007) and Preller et al. (2016). Table 1 contains the information on all data included in the meta-analysis. Several articles reported data from repeated measurements with varying psilocybin doses, so we treated these data as statistically dependent observations. Corresponding information on data dependencies was added to the datasets.

**Table 1.** Summary of studies included in the meta-regression analysis. All studies were performed with healthy study participants. Two studies report the data of the same sample (included only once in the meta-analysis) and two studies report subsamples of data already included in a meta-analysis, which were therefore omitted. Several studies contain multiple observations (e.g. from repeated measurements).

Study	Sample Size	Study description	Data report	Psilocybin administration
Umbricht et al., 2002	*N* = 18	EEG, auditory mismatch-negativity paradigm	5D-ASC (converted)	Oral administration Dosage: (1) 280 μg/kg body weight
Hasler et al., 2004	*N* = 8	Double-blind placebo-controlled within-subject design ECG, blood pressure, body temperature and further questionnaire assessment	5D-ASC	Oral administration as gelatin capsules Dosages: (1) 45 μg/kg body weight (2) 115 μg/kg body weight (3) 215 μg/kg body weight (4) 315 μg/kg body weight
Carter, Pettigrew, et al., 2005 (Same sample: Wittmann et al., 2007)	*N* = 12 (*N* = 12)	Double-blind placebo-controlled within-subject design Binocular rivalry paradigm, time reproduction, auditory sensorimotor synchronization task, finger tapping, spatial span test	5D-ASC	Oral administration as gelatin capsules Dosages: (1) 115 μg/kg body weight (2) 250 μg/kg body weight
Carter et al., 2007 (Subsample: Carter, Burr, et al., 2005)	*N* = 10 (*N* = 8)	Within-subject design with additional conditions: (2) placebo, (3) 50 mg ketanserin, (4) ketanserin + psilocybin, Multiple object tracking task, spatial working memory task	5D-ASC	Oral administration as gelatin capsules Dosage: (1) 215 μg/kg body weight
Vollenweider et al., 2007	*N* = 16	Double-blind placebo-controlled within-subject design Prepulse inhibition of acoustic startle response	5D-ASC (converted)	Oral administration as gelatin capsules Dosages: (1) 115 μg/kg body weight (2) 215 μg/kg body weight (3) 315 μg/kg body weight
Quednow et al., 2012	*N* = 16	Double-blind within-subject design with additional conditions: (2) placebo, (3) 40 mg ketanserin, (4) ketanserin + psilocybin, Prepulse inhibition of acoustic startle response, colour-word-Stroop test	5D-ASC (converted)	Oral administration as gelatin capsules Dosage: (1) 260 μg/kg body weight
Pokorny et al., 2016	*N* = 19 *N* = 17	Double-blind within-subject design with two groups (G1: *N* = 19, G2: *N* = 17) and additional conditions: (2) G1/G2: placebo, (3) G1: 20 mg buspirone, or G2: 3 mg ergotamine, (4) G1: buspirone + psilocybin, or G2: ergotamine + psilocybin	5D-ASC	Oral administration as gelatin capsules Dosage: (1) 170 μg/kg body weight (G1) (2) 170 μg/kg body weight (G2)
Kometer et al., 2012	*N* = 17	Double-blind placebo-controlled within-subject design EEG, facial emotional recognition task, emotional Go/NoGo task	11-ASC	Oral administration as gelatin capsules Dosage: (1) 215 μg/kg body weight
Schmidt et al., 2013 (Same sample: Schmidt et al., 2012)	*N* = 21 (*N* = 20)	Double-blind placebo-controlled within-subject design with additional condition: (2) S-ketamine EEG, auditory mismatch-negativity paradigm, backward masking paradigm with facial affect discrimination	11-ASC	Oral administration as gelatin capsules Dosage: (1) 115 μg/kg body weight
Bernasconi et al., 2014	*N* = 30	Placebo-controlled EEG, passive viewing of emotional face task	11-ASC	Oral administration as gelatin capsules Dosage: (1) 170 μg/kg body weight
Pokorny et al., 2017 (Subsample: Preller et al., 2016)	*N* = 33 (*N* = 21)	Double-blind placebo-controlled within-subject design Multifaceted empathy test, moral dilemma task ,fMRI, Cyberball task	11-ASC	Oral administration as gelatin capsules Dosage: (1) 215 μg/kg body weight
Lewis et al., 2020	*N* = 55	Randomized double-blind placebo-controlled, repeated measures design	11-ASC	Oral administration Dosages: (1) 160 ug/kg body weight (2) 215 ug/kg body weight
Smigielski et al., 2020	*N* = 17	Double-blind placebo-controlled within-subject crossover design, EEG, self-monitoring task	11-ASC	Oral administration as capsules Dosage:
				(1) 230 ug/kg body weight
Carbonaro et al., 2018	*N* = 20	Double-blind placebo-controlled within-subject design with additional conditions: (4) 400 mg/70 kg dextromethorphan Blood pressure, heart rate, pupil diameter, circular lights, balance, repeated administration of other questionnaires	11-ASC HRS MEQ30	Oral administration as gelatin capsules Dosages: (1) 143 μg/kg body weight (10 mg/70 kg) (2) 286 μg/kg body weight (20 mg/70 kg) (3) 429 μg/kg body weight (30 mg/70 kg)
Griffiths et al., 2006 (reported in Barrett et al., 2015)	*N* = 30	Double-blind within-subject design with additional conditions (data from unblended control condition not included): (2) 40 mg/70 kg body weight Methylphenidate Group setting, meetings with monitor before and after sessions	HRS MEQ30	Oral administration Dosage: (1) 429 μg/kg body weight (30 mg/70 kg)
Griffiths et al., 2011 (reported in Barrett et al., 2015)	*N* = 18	Double-blind placebo-controlled between-group crossover design Descending or ascending dosage order Group setting, meetings with monitor before and after sessions	HRS MEQ30	Oral administration as gelatin capsules Dosages: (1) 71 μg/kg body weight (5 mg/70 kg) (2) 143 μg/kg body weight (10 mg/70 kg) (3) 286 μg/kg body weight (20 mg/70 kg) (4) 429 μg/kg body weight (30 mg/70 kg)
Nicholas et al., 2018	*N* = 12	Lying on sofa with eye-shades headphones and music Preparation of participants with guides Blood sample, ECG	MEQ30	Oral administration as capsules Dosages (given in escalating order): (1) 300 μg/kg body weight (2) 450 μg/kg body weight (3) 600 μg/kg body weight

EEG: electroencephalogram; ECG: electrocardiogram; fMRI: functional magnetic resonance imaging.

### Questionnaires

Our meta-analysis included data from three different questionnaires commonly applied in research on the subjective experiences induced by psychedelic substances: the Altered States of Consciousness Rating Scale (5D-ASC; Dittrich et al., 2006, 2010), the Mystical Experience Questionnaire (MEQ30; Pahnke, 1963, 1966) and the Hallucinogen Rating Scale (HRS; Strassman et al., 1994).

The 5D-ASC has become one of the most frequently used psychometric tools in the assessment of ASC. It is designed to investigate the characteristics of ASC that are invariant across various methods that are used to induce ASC, including both pharmacological (e.g. psilocybin, mescaline, ketamine) and non-pharmacological ones (e.g. sensory deprivation, hypnosis, autogenic training). Over the course of more than 30 years, the questionnaire has undergone several refinements finally leading to the currently used version that comprises 94 items (Dittrich et al., 2006, 2010). Two different ways to analyse the questionnaire are in use. The first is referred to as 5D-ASC, where the ratings of 66 items are combined to form three core dimensions: (1) *Oceanic Boundlessness*, (2) *Dread of Ego Dissolution* and (3) *Visionary Restructuralization*. Based on the remaining 28 items, the analysis is supplemented with two empirically derived scales that are considered specific to certain induction methods: (4) *Auditory Alterations* and (5) *Vigilance Reduction* (Dittrich et al., 2006, 2010). The second way of analysis uses only 42 items of the three core dimensions (Studerus et al., 2010) and summarizes the item scores along 11 factors (11-ASC)., These factors can be correspondingly considered subscales of the three core dimensions and have been termed (1) *Experience of Unity*, (2) *Spiritual Experience*, (3) *Blissful State*, (4) *Insightfulness*, (5) *Disembodiment*, (6) *Impaired Control and Cognition*, (7) *Anxiety*, (8) *Complex Imagery*, (9) *Elementary Imagery*, (10) *Audio-Visual Synesthesia*, and (11) *Changed Meaning of Percepts*. Both analysis schemes have been validated and demonstrate good reliability ((5D-ASC: Hoyt 0.88–0.95; Dittrich et al., 2006, 2010); 11-ASC: mean Cronbach’s alpha of 0.83 (Studerus et al., 2010)).

The initial MEQ was first used in the famous ‘Good Friday Experiment’ (Pahnke, 1963, 1966), where it was intended to assess the differences regarding the aspects of mystical experience between a group taking psilocybin and a control group taking a placebo. The items of the MEQ were chosen based on literature about mysticism including first-person accounts as well as theoretical work, most notably by James (1906) and Stace (1960). The initial MEQ has been further developed; the most recent is a condensed version MEQ30 by MacLean et al. (2012), consisting of 30 items and four empirical scales: (1) *Sacredness*, (2) *Positive Mood*, (3) *Transcendence of Time/Space*, and (4) *Ineffability*. This factor structure is currently recommended for analyses and has been assessed for reliability, yielding very good scores for all four subscales (Cronbach’s alpha: 0.80 to 0.95) (Barrett and Griffiths, 2017; Barrett et al., 2015).

Originally developed to quantify acute effects of imethyltryptamine (DMT; Strassman et al., 1994), the HRS has become a frequently used instrument in the assessment of hallucinogen-induced ASC. The initial construction of this questionnaire was based on systematic interviews with experienced hallucinogen users describing the effects of smoking DMT freebase. The effects specifically induced by DMT, as well as general characteristic effects of hallucinogenic substances, were intended to be covered by the resulting collection of items. The HRS measures six conceptually distinct dimensions of ASC that were a priori defined and referred to as ‘clinical clusters’: (1) *Somaesthesia*: interoceptive, visceral, and cutaneous/tactile effects, (2) *Affect*: emotional/affective responses, (3) *Perception*: visual, auditory, gustatory, and olfactory experiences, (4) *Cognition*: alterations in thought processes or content, (5) *Volition*: a change in capacity to willfully interact with oneself, the environment, or certain aspects of the experience, and (6) *Intensity*: the overall strength and course of the experience (Strassman et al., 1994). Revision and refinement of the early versions finally resulted in the HRS 3.06 as the most recent version (available from the questionnaire’s author upon request), containing 100 statements, most of which are rated on a 5-point Likert scale. Reliability assessment indicates good internal consistency for the *Affect, Somaesthesia, Cognition* and *Perception* scales (Riba et al., 2001).

### Standardization of data

Some studies reported the dose normalized to body weight (e.g. 25 mg per 70 kg body weight). To allow regression analyses, we converted the doses to microgram (10^−6^ g) per kilogram body weight.

With regards to the psychometric data, some studies (Quednow et al., 2012; Umbricht et al., 2002; Vollenweider et al., 2007; Wittmann et al., 2007) reported the scores of the 5D-ASC as non-normalized sums of item scores. These scores were converted to the ‘percentage of maximum score’, in line with the ASDB (Schmidt, 2017).

### Statistical analyses

We performed linear meta-regression analyses for each factor and scale of the respective questionnaire. Although dose–response relationships are usually best described by a sigmoid function, the available data did not cover the upper and lower bounds to resemble a sigmoid function. We, therefore, modelled a linear dose–response relationship, which constitutes a suitable approximation for the dynamic range of a sigmoid function. We used a random effects model to estimate the true underlying effects across studies (Borenstein et al., 2007), namely the intercept and slope of the dose–response function. A random effects model was employed to consider between-study variance, which can result from, for example, differences in participant characteristics, interventions, the state of mind of participants (set), and the environment of substance intake (setting) across studies (Zinberg, 1984). Multiple studies used a within-subject design in which different doses were administered to the same sample of study participants. To account for these statistically dependent effect sizes, we used the RVE framework developed by Hedges et al. (2010) with small sample adjustment by Tipton (2015). The RVE framework permits the inclusion of multiple effect size estimates from a study without the knowledge of the underlying covariance structure by assuming a common correlation *p* (0–1) between within-study effect sizes (*p* = .8 was used as the recommended default value (Tanner-Smith and Tipton, 2014)). To test whether the choice of *p* affected the obtained parameter estimates, we performed a sensitivity analysis. The weights were calculated using the correlated effects model with the inverse of the sampling variance in combination with a method of moments estimator by Hedges et al. (2010). Heterogeneity was assessed by estimating the degree of inconsistency across studies using *I*² (Borenstein et al., 2017; Higgins and Thompson, 2002) and the between-study variance with Tau² (Deeks et al., 2011). Analyses were performed using the robumeta package (Fisher and Tipton, 2015) in R version 3.6.2 (R Core Team, 2019).

To allow comparability with previous reports, we used spiderplots for the visualization of the results for all questionnaires, inspired by the reports of Vollenweider and Kometer (2010) and Bayne et al. (2016). Spiderplots provide an overview of all questionnaire factors and scales by showing the percentage of the maximum score for different doses calculated with the linear regression estimates. In addition, we present dose–response relationships for each factor and scale, including the effect sizes of the individual studies presented as circles. The size of a circle represents the magnitude of the calculated weight of a study sample. We generated spiderplots with the fmsb package (Nakazawa, 2019) and scatterplots with the plot function in R version 3.6.2 (R Core Team, 2019).

### Additional meta-analysis including patient data

In addition to the main analysis, which comprises only data on healthy study participants, we performed a second analysis. This additional analysis was performed identically, however, patient data were added.

As patient data were not systematically included in the ASDB, we performed a literature search in Google Scholar (as described in Schmidt and Berkemeyer, 2018) and a hand-search in reference lists of thematically relevant literature to identify additional patient datasets published until 20 December 2020. This search strategy identified one study reporting data on patients with cancer-related psychiatric distress (Ross et al., 2016), one study reporting data on patients with major depressive disorder (Davis et al., 2020) and two studies reporting data on patients with treatment-resistant major depression (Carhart-Harris et al., 2016, 2018). However, the data in Carhart-Harris et al. (2016) constitute a subsample of Carhart-Harris et al. (2018). Therefore, we included only the data from Carhart-Harris et al. (2018) in the 11-ASC analysis. The available ASDB data on patients with alcohol use disorder (Bogenschutz et al., 2015) and with cancer-related psychiatric distress (Griffiths et al., 2016; Grob et al., 2011) were included in the 5D-ASC analysis. The additional analysis on the MEQ30 included data on patients with cancer-related psychiatric distress (Griffiths et al., 2016; Ross et al., 2016) and major depressive disorder (Davis et al., 2020). The additional analysis on the HRS included data on patients with cancer-related psychiatric distress (Griffiths et al., 2016). The reported doses in Griffiths et al. (2016) were given for a range instead of a specific dose (1–3 mg per 70 kg; 22 or 30 mg per 70 kg). Therefore, we performed the analyses with the transformed weighted mean of the respective doses (21 μg/kg and 317 μg/kg body weight), which was calculated based on the number of patients receiving each dose. The data in Carhart-Harris et al. (2018) were not adjusted for body weight, so we assumed an average body weight of 70 kg and adjusted the reported doses accordingly. Supplementary Table 1 specifies the studies that were additionally included in this analysis.

## Results

### Data description

We included psychometric data from 17 studies in which psilocybin was orally administered to healthy study participants. Table 1 summarizes all data included in the meta-analysis. As several studies report repeated measurements, the final dataset comprised 14 observations from 7 samples of participants for the 5D-ASC (except for the *Auditory Alterations* and *Vigilance Reduction* scales with 12 observations from 5 samples); 8 observations from 6 samples for the 11-ASC (except for the scales *Blissful State, Experience of Unity, Insightfulness* and *Spiritual Experience* with 10 outcomes from 7 samples); 11 observations from 4 samples for the MEQ30; and 8 observations from 3 samples for the HRS.

### Dose–response relationships

**Figure 1.**
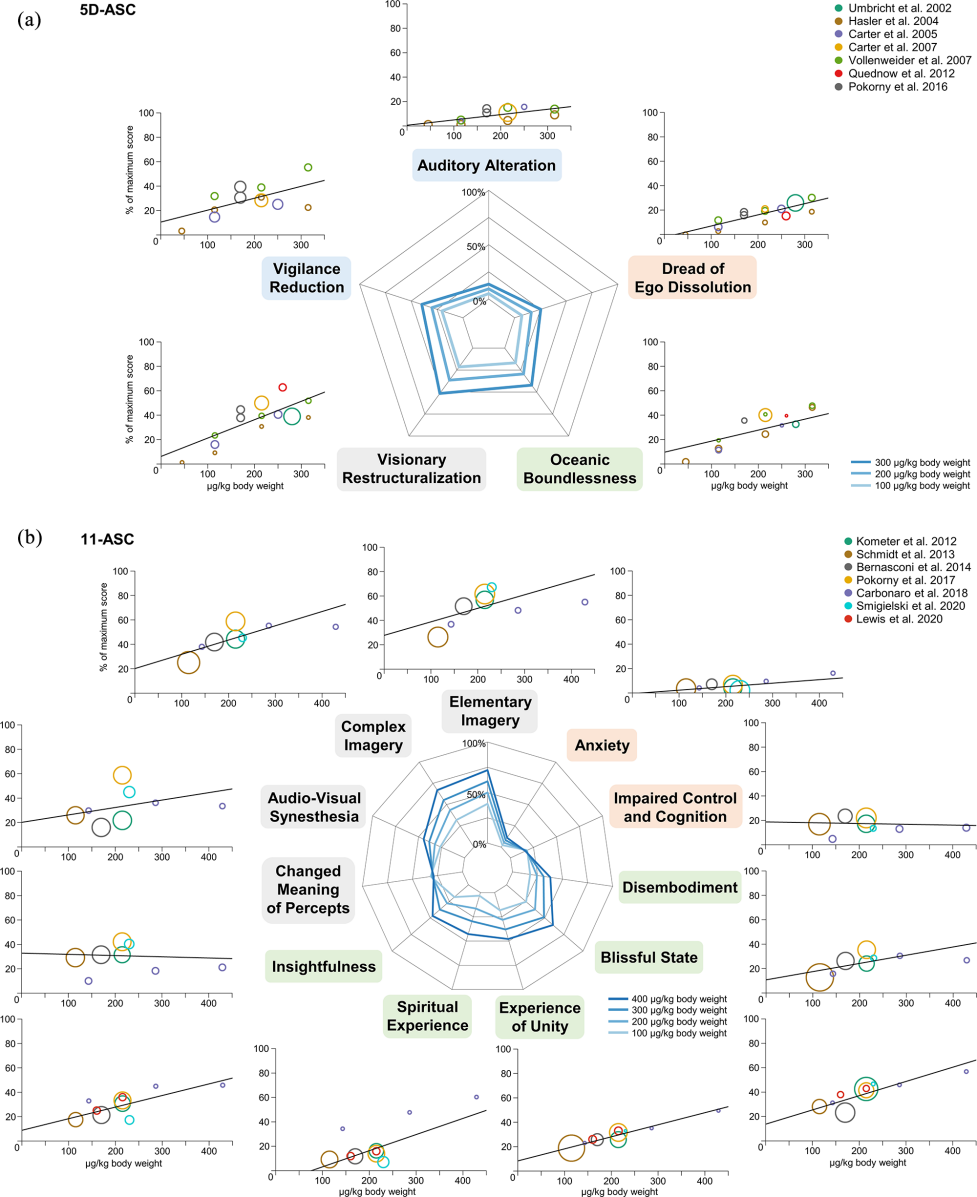
Dose–response relationships for the Altered States of Consciousness Rating Scale. (a) Dose-specific subjective effects of psilocybin measured with the Altered States of Consciousness Rating Scale. The data of this instrument can be analysed according to a schema where items are organized into five factors, called ‘dimensions’ of ASC experiences (5D-ASC). (b) A finer-grained quantification of specific aspects of subjective experiences is obtained when the questionnaire is analysed according to the 11-factors schema. These 11 factors can be considered subscales of the three core dimensions of the 5D-ASC, namely *Oceanic Boundlessness, Dread of Ego Dissolution* and *Visionary Restructuralization* (see corresponding colours of the subscale names). Doses are given in microgram per kilogram body weight; effects are given as the percentage score of the maximum score on each factor (questionnaire items are anchored by 0% for ‘No, not more than usual’ and 100% for ‘Yes, much more than usual’). Circle colour indicates data from the same sample of participants (the same colour corresponds to statistically dependent data), while circle size represents the weight of the data based on study variance (see Methods). Spiderplots present the estimated dose–responses for 100–300 μg/kg body weight on the 5D-ASC and 100–400 μg/kg body weight on the 11 subscales, corresponding to the range of doses that were included in the respective analyses. The colour of individual scales corresponds to the primary dimensions and the respective subscales.

**Figure 2.**
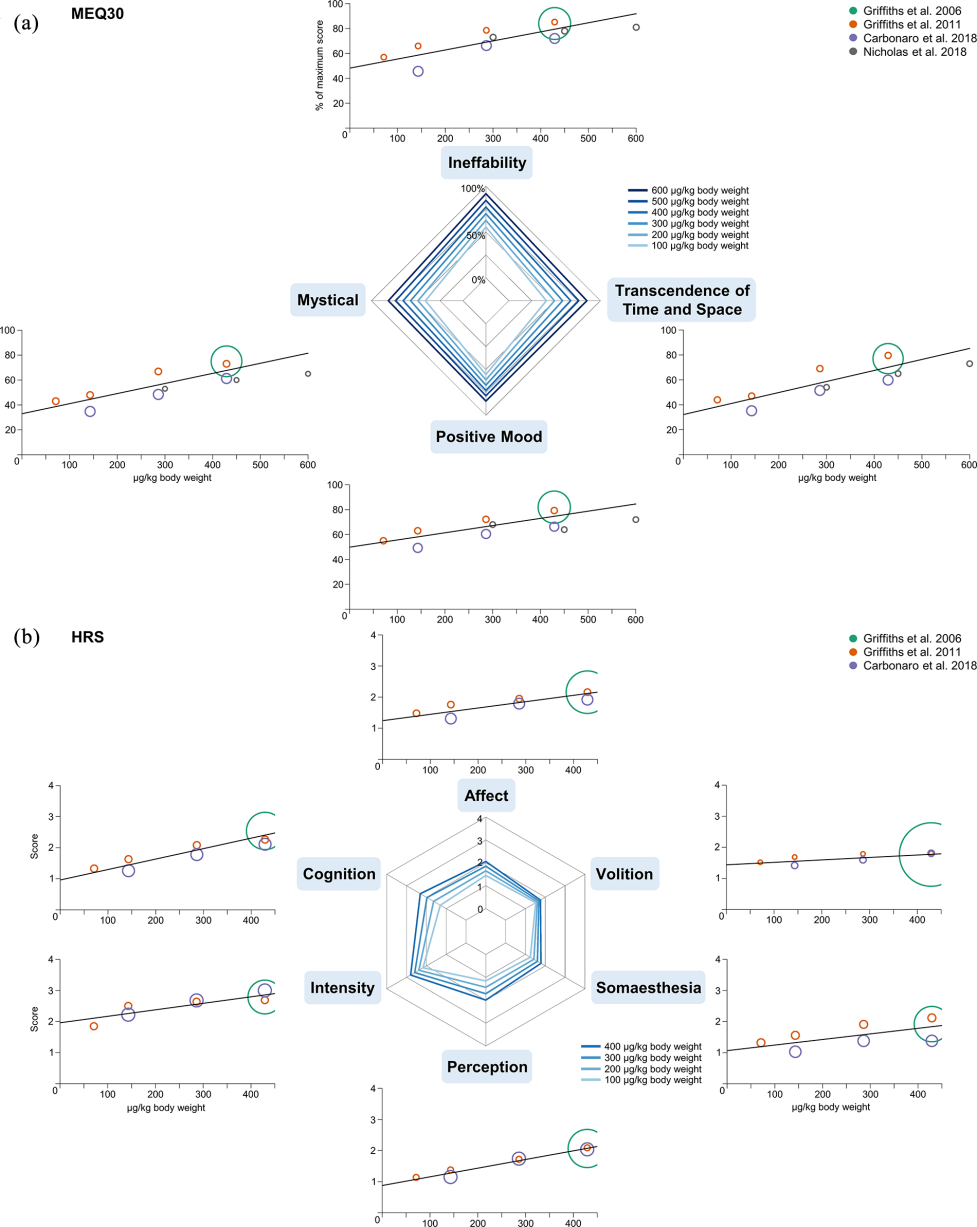
Dose–response relationships for MEQ30 and HRS. Dose-specific subjective effects of psilocybin for the psychometric instruments (a) MEQ30 and (b) HRS. Doses are given as microgram per kilogram body weight. Effects on the MEQ30 are presented as the percentage score of the maximum score. Effects on the HRS range from 0 to 4 (items in the questionnaire from 0 ‘Not at all’ to 4 ‘Extreme’). Circle colour indicates data from the same sample of participants (the same colour corresponds to statistically dependent data), circle size represents the weight of the data based on study variance (see Methods). Spiderplots present the estimated dose–responses for 100–600 μg/kg body weight on the MEQ30 and 100–400 μg/kg body weight on the HRS, corresponding to the range of doses that were included in the respective analyses.

Regression coefficients for the dose–response analyses and heterogeneity parameters are summarized in Table 2. Ratings on all factors and scales of the included questionnaires positively correlated with the psilocybin dose, except for the 11-ASC subscales *Changed Meaning of Percept* and *Impaired Control and Cognition*. Spiderplots for each questionnaire and the dose–response relationships for each factor and scale of the respective questionnaires are presented in Figure 1 (5D-ASC and the subscales of the ASC rating scale) and Figure 2 (MEQ30 and HRS).

**Table 2.** Meta-regression estimates for all included questionnaires with respective factors/dimensions/subscales. Coefficients (Coeff.) are presented with 95% confidence intervals (CIs) and standard errors (SE). The *t*-test statistic determines whether a linear relationship exists under the null hypothesis that the slope is equal to zero. Tau² indicates the between-study variance and *I*² indicates the degree of inconsistency across studies in percent. Intercept estimates are rounded to the first decimal, except for the HRS due to its different range (0–4). Slope estimates are rounded to the third decimal considering its greater sensitivity to increasing dose.

Outcome	Intercept			Slope			*t* (df)	*p*	Tau²	*I*²
	Coeff.	(95% CI)	SE	Coeff.	(95% CI)	SE				
5D-ASC										
Auditory Alterations	0.6	(–8.7−9.9)	1.71	0.044	(0.006−0.081)	0.0062	7.1 (1.5)	.040	4.2	22.3
Oceanic Boundlessness	4.0	(–28.9−37.0)	9.67	0.127	(0.005−0.249)	0.0388	3.3 (3.1)	.045	30.8	43.9
Dread of Ego Dissolution	–2.2	(–10.4−6.0)	2.40	0.092	(0.062−0.122)	0.0093	9.8 (3.0)	.002	0.0	0.0
Vigilance Reduction	10.6	(–6.4−27.5)	4.15	0.098	(–0.015−0.211)	0.0252	3.9 (1.9)	.065	78.7	64.0
Visionary Restructuralization	6.3	(–22.3–34.9)	9.06	0.151	(0.032−0.269)	0.0390	3.9 (3.3)	.026	71.4	65.3
11-ASC										
Anxiety	–0.5	(–27.8–26.7)	3.69	0.029	(–0.121−0.179)	0.0203	1.4 (1.3)	.348	3.9	49.0
Audio-Visual Synesthesia	20.2	(–66.4–106.8)	13.89	0.061	(–0.449−0.571)	0.0692	0.9 (1.3)	.510	243.7	83.3
Blissful State	13.8	(–27.0–54.6)	7.91	0.117	(–0.091−0.324)	0.0352	3.3 (1.5)	.115	18.2	40.1
Complex Imagery	20.2	(–91.9–132.2)	16.16	0.117	(–0.480−0.713)	0.0796	1.5 (1.3)	.338	61.2	59.2
Changed Meaning of Percepts	32.8	(–3.2–68.8)	5.70	–0.010	(–0.352−0.332)	0.0432	–0.2 (1.3)	.850	114.1	79.0
Disembodiment	10.8	(–79.1–100.8)	12.02	0.067	(–0.401−0.536)	0.0611	1.1 (1.3)	.435	28.7	43.2
Elementary Imagery	27.8	(–139.6–195.1)	24.94	0.111	(–0.873−1.090)	0.1270	0.9 (1.3)	.517	141.7	76.2
Experience of Unity	8.3	(–3.7–20.4)	1.86	0.099	(0.038−0.160)	0.0103	9.6 (1.5)	.025	0.0	0.0
Insightfulness	8.8	(–10.4–28.1)	3.80	0.095	(–0.006−0.197)	0.0172	5.5 (1.5)	.055	9.2	22.6
Impaired Control & Cognition	18.7	(1.6–35.8)	2.41	–0.007	(–0.093−0.070)	0.0089	–0.7 (1.2)	.579	21.1	58.2
Spiritual Experience	–10.0	(–63.4–43.4)	10.09	0.132	(–0.224−0.488)	0.0632	2.1 (1.6)	.205	42.8	72.4
MEQ30										
Ineffability	48.2	(–13.1−109.5)	10.75	0.073	(–0.020−0.166)	0.0243	3.0 (2.3)	.081	45.3	59.0
Mystical	32.9	(–28.4−94.3)	9.90	0.081	(–0.021−0.183)	0.0264	3.1 (2.3)	.078	58.1	58.4
Positive Mood	49.8	(2.7−97.0)	7.97	0.058	(–0.034−0.150)	0.0239	2.4 (2.3)	.122	62.2	70.1
Transcendence of Time & Space	32.2	(–22.5−86.8)	9.23	0.090	(0.002−0.175)	0.0227	3.9 (2.3)	.048	77.5	72.0
HRS										
Affect	1.24	(–1.08−3.57)	0.236	0.002	(–0.001−0.005)	0.0005	3.9 (1.7)	.078	0.01	41.4
Cognition	0.96	(–1.56−3.48)	0.261	0.003	(0.000−0.007)	0.0007	4.5 (1.7)	.059	0.03	50.1
Intensity	1.96	(1.58−2.34)	0.040	0.002	(0.001−0.003)	0.0003	7.8 (1.7)	.024	0.02	50.3
Perception	0.88	(–0.20−1.95)	0.112	0.003	(0.001−0.004)	0.0003	10.6 (1.7)	.015	0.00	0.0
Somaesthesia	1.07	(–1.94−4.08)	0.313	0.002	(–0.001−0.005)	0.0006	3.1 (1.7)	.107	0.09	83.9
Volition	1.44	(–0.23−3.11)	0.154	0.001	(–0.001−0.003)	0.0004	2.2 (1.5)	.200	0.00	0.0

### Sensitivity analyses

To test the robustness of the estimated RVE parameters (intercept and slope), we examined whether the estimates were stable for different values of *p* (0–1) (see Methods). Across all analyses, intercept parameters differed only in the range of 0 to 0.18, and slope parameters were virtually identical, differing only in the range of 0 to 0.0007. Therefore, in line with (Tipton, 2015), the sensitivity analyses produced robust effect size estimates for different values of *p*.

### Additional meta-analysis including patient data

In the additional analysis, we included the available patient data on the 5D-ASC (Bogenschutz et al., 2015; Griffiths et al., 2016; Grob et al., 2011;), 11-ASC (Carhart-Harris et al., 2018), MEQ30 (Davis et al., 2020; Griffiths et al., 2016; Ross et al., 2016) and HRS (Griffiths et al., 2016). The meta-regression estimates on all factors and scales were similar to the analyses on healthy study participants (see Supplemental material).

## Discussion

Here we performed a meta-analysis on psychometric data to estimate linear dose–response relationships for psilocybin-induced subjective experiences assessed with standardized questionnaires. Our analyses revealed positive correlations of effects and doses for most factors and scales of the questionnaires tested.

For the 5D-ASC questionnaire, we found the strongest dose–responses for the scales *Visionary Restructuralization*, comprising alterations in perception, and *Oceanic Boundlessness*, comprising positively experienced ego dissolution, that is, derealization and depersonalization associated with positive affect, ranging from heightened mood to euphoric exaltation. Interestingly, a medium dose–response was found for *Vigilance Reduction*, relating to states of drowsiness, reduced alertness and impaired cognitive function. Since classic psychedelics like psilocybin are usually characterized by a lack of sedation and clouding of consciousness, it was suggested that the effect of psilocybin on *Vigilance Reduction* rather reflects the state of contemplativeness, dreaminess and a reduction in attentiveness (Studerus et al., 2011). *Dread of Ego Dissolution* associated with loss of self-control and anxiety exhibited a small dose–response with comparatively low rating scores. *Auditory Alterations*, relating to acoustic hallucinations and distortions in auditory experiences, were barely experienced. Analysis of the 11-ASC reflected the finer facets of subjective experiences. Consistent with the 5D-ASC, the strongest dose–responses were found for subscales referring to *Visionary Restructuralization*, that is, *Elementary*- and *Complex Imagery.* In contrast, *Audio-Visual Synesthesia* and *Changed Meaning of Percepts* exhibited little modulation by dose. The intensity of the subscales referring to *Oceanic Boundlessness* increased with dose, especially for *Spiritual Experience* and *Blissful State*. In contrast, subscales referring to *Dread of Ego Dissolution* were barely modulated by dose and exhibited only small effects.

The MEQ30 questionnaire aims to measure different aspects of mystical-type experiences. The effects of psilocybin were characterized by relatively large and similar effects on all four factors of the questionnaire. It had been suggested that scores >60% on each of the four factors indicate a complete mystical experience (Barrett et al., 2015). According to the obtained estimates, such experiences are expected for doses of approximately 350 μg/kg body weight and above. Interestingly, even very small doses induced relatively high scores, and the fitted regression lines had a relatively high y-axis intercept. While responses for mid-range doses are covered well by the available data, the effects for doses in the lower range could not be clearly established.

On the HRS questionnaire, the dimensions *Cognition* and *Perception* showed the largest response, whereas *Volition* was barely modulated by dose.

In summary, psilocybin mainly induced dose-dependent alterations in perception and positively experienced ego dissolution. Subjective experiences for high doses of psilocybin are characterized by all aspects of mystical-type experiences captured by the MEQ30 questionnaire. The given data did not support the premise that higher doses of psilocybin would directly induce the more aversive aspects of experiences; however, as the given data are average scores, it cannot be excluded that some individuals undergo highly challenging experiences.

### Statistical power and heterogeneity

To use the estimated linear dose–response models for predictions of psilocybin responses in future studies, it is important to discuss the robustness of the obtained estimates. Assessing the power of the given meta-regression approach is challenging due to the dependencies in the data structure. Hempel et al. (2013) consider heterogeneity to be the most important factor in assessing power in meta-analyses. Jenkins and Quintana-Ascencio (2020) suggest that eight studies is the minimum amount for meta-regressions to be informative when the variance is small and 25 when variance is large. With 8 to 14 observations, the amount of available data in the present analysis was at the lower end of the recommended range. The between-study variance, Tau², was rather small for most of the factors and scales, indicating that the obtained estimates are reliable (see Table 2). The degree of inconsistency across studies, *I*², represents the proportion of the observed variance that is due to systematic differences between studies rather than random error, for example, resulting from differences in study population, study design or bias (Borenstein et al., 2017; Higgins and Thompson, 2002). Values between 0% and 60% indicate a small to moderate degree of inconsistency (Deeks et al., 2011), which was the case for most factors and scales in our analysis. However, we found considerable inconsistencies (*I*² > 75%) for the scales *Audio-Visual Synesthesia, Changed Meaning of Percepts* and *Elementary Imagery* on the 11-ASC as well as *Somaesthesia* on the HRS. Until more data are available for these scales, corresponding dose–response estimates need to be treated with caution and confidence intervals should be considered. Taken together, our analysis provided robust dose–response relationships for most factors and scales.

### Non-pharmacological influences on subjective experiences

Although the psilocybin dose is the most important determinant of the acute psychedelic experience, there is considerable inter- and intra-individual variability in subjective responses to psilocybin (Haijen et al., 2018; Russ et al., 2019a, 2019b; Studerus et al., 2012). A fundamental concept in psychedelic research is that the subjective experience is determined by the interaction of substance, *set* and *setting* (Hartogsohn, 2017; Leary et al., 1963; Zinberg, 1984). *Set* constitutes the personality of the substance user and the preparation, expectation and intention of substance use (Leary et al., 1963; Zinberg, 1984), whereas *setting* refers to the physical, social and cultural environment of substance administration (Leary et al., 1963; Zinberg, 1984). Included studies generally adhered to guidelines to minimize adverse effects by providing adequate selection and preparation of study participants and a suitable setting for psilocybin administration (Fischman and Johanson, 1998; Johnson et al., 2008). While this ensures comparability to some degree, non-pharmacological variables were not standardized, nor systematically assessed, and potentially varied, making them worth discussing.

Multiple set-related factors have been identified as affecting the quality of the subjective psilocybin experience. First and foremost, current mood, psychological distress prior to psilocybin administration and clear intentions have been shown to exert influence (Dittrich, 1994; Metzner et al., 1965; Studerus et al., 2012). Second, effects of personality traits have been reported: trait absorption was shown to promote all aspects of subjective experience (Haijen et al., 2018; Studerus et al., 2012), extroversion has been associated with visionary experiences (Dittrich, 1994; Smigielski et al., 2019a; Studerus et al., 2012), openness (Dittrich, 1994; MacLean et al., 2011; Smigielski et al., 2019a), optimism towards life (Smigielski et al., 2019a) and being in a state of surrender (Russ et al., 2019a, 2019b) have been associated with peak or mystical-type experiences. The trait neuroticism has been reported to increase the occurrence of challenging experiences in some studies (Barrett et al., 2017; Dittrich, 1994; Hemsley and Ward, 1985), while other studies did not find such an association (Haijen et al., 2018; Smigielski et al., 2019a; Studerus et al., 2012). In addition, preoccupation (Russ et al., 2019a, 2019b) and emotional excitability prior to the experience (Studerus et al., 2012) have been associated with challenging experiences, whereas feeling well prepared, having a recreational intention and emotional reappraisal have been shown to reduce the occurrence of challenging experiences (Haijen et al., 2018; Smigielski et al., 2019a).

The setting in laboratory experiments is typically strongly controlled (Fischman and Johanson, 1998; Johnson et al., 2008), but the degree of interpersonal support, the amount and difficulty of tasks performed and the general ambience potentially varied across the included studies. Previous work indicated that spatially confined neuroimaging settings can increase the likelihood of challenging experiences (Studerus et al., 2012). However, the only study in our analysis (11-ASC) using magnetic resonance imaging (Pokorny et al., 2017) reported similar effects compared with the other studies on scales referring to challenging experiences: *Anxiety* and *Impaired Control and Cognition*.

Some additional factors not covered by the concepts of set and setting may contribute to the variability of experiences. First, age may play a role. Several studies reported that older study participants experienced less *Impaired Control and Cognition* and tend to experience more of a *Blissful State* compared with younger study participants, whereas younger study participants more often report challenging experiences (Dittrich, 1994; Hyde, 1960; Metzner et al., 1965; Studerus et al., 2012). Second, the amount of previous experience with psychedelics may have an influence on the current experience. Hallucinogen-naïve study participants reported slightly more *Visionary Restructuralization, Disembodiment*, and *Changed Meaning of Percepts* compared with experienced psilocybin users (Metzner et al., 1965; Studerus et al., 2012). Further, differences in individual pharmacokinetics were reported in terms of plasma psilocin levels and 5-HT2AR occupancy, which were found to correlate with the overall subjective experience (Brown et al., 2017; Hasler et al., 2004; Lindenblatt et al., 1998; Madsen et al., 2020). Finally, brain structure metrics have been reported to correlate with experiences, that is, a correlation of the thickness of the rostral anterior cingulate and subscales of 5D-ASC dimension *Oceanic Boundlessness* (Lewis et al., 2020).

In sum, psilocybin dose can be considered the most important determinant of subjective experiences. However, it should be noted that studies reported relatively large proportions of unexplained variance in subjective responses to psilocybin (Haijen et al., 2018; Studerus et al., 2012). The potential influence of non-pharmacological factors must not be underrated and warrants consideration when interpreting dose–response relationships. Future research could benefit from more standardized assessment of non-pharmacological factors in order to evaluate their effect on subjective experiences with meta-analytical approaches.

### Comparison with subjective experiences of patients

We performed an additional analysis to test whether the health status of study participants may also influence the reported experiences. To this end, we included patient data on the 5D-ASC, 11-ASC, MEQ30 and HRS in the analysis (see Supplemental material). Patient ratings were comparable to those of healthy study participants on all questionnaires. On the 5D-ASC, the ratings of patients with alcohol use disorder and cancer-related psychiatric distress were found to be slightly higher for *Oceanic Boundlessness* and slightly lower for *Dread of Ego Dissolution*. This was consistent with the ratings on the 11-ASC, where patients with treatment-resistant major depression rated slightly higher on the subscales of *Oceanic Boundlessness* (*Blissful State, Disembodiment, Experience of Unity, Insightfulness, Spiritual Experience*). However, patient ratings were higher on the subscale *Anxiety.* (Note: Data also indicated large within-study variance for this subscale.) While more studies are needed to confirm these differences, it can also be speculated that the differences may result from the fact that these studies were designed to facilitate peak or mystical-type experiences, which were reported to mediate therapeutic outcomes (Agin-Liebes et al., 2020; Bogenschutz et al., 2015; Carhart-Harris et al., 2016; Carhart-Harris et al., 2018; Davis et al., 2020; Garcia-Romeu et al., 2015; Griffiths et al., 2016; Roseman et al., 2018; Ross et al., 2016).

### Comparison with previous dose–response reports

So far, the number of studies reporting data to approximate dose–response relationships for psilocybin is small and the included data were limited to data assessed by the same research groups. Vollenweider and Kometer (2010) and Studerus et al. (2011) previously reported approximately linear dose–response relationships for subjective experiences assessed with the 5D-ASC and 11-ASC. In line with our results, they found the strongest effects for perceptual alterations, followed by the subscales referring to *Oceanic Boundlessness*. Contrary to the results of our meta-analysis across multiple studies from different research groups, they reported stronger effects for *Audio-Visual Synesthesia* and smaller effects for *Spiritual Experience*. They also found dose-dependent effects for the 11-ASC subscales *Changed Meaning of Percepts* and *Impaired Control and Cognition*, whereas we did not find evidence for dose-dependent effects. In our meta-analysis, the heterogeneity parameter, *I*², indicated considerable inconsistencies for those subscales. Therefore, the differences may be the result of methodological differences between studies, that is, with regards to the amount and difficulty of tasks performed.

### Comparison with intravenous application of psilocybin and combination with other treatments

Since we only included studies where psilocybin was administered orally, we excluded one study with intravenous administration of 1.5 mg and 2 mg (Carhart-Harris et al., 2011). Despite the considerably smaller dose quantities, the pattern of responses on the 5D-ASC were roughly comparable to our results, corresponding to a low oral dose (i.e. 100–150 ug/kg body weight) for 1.5 mg intravenously and a medium oral dose (i.e. 200–250 ug/kg body weight) for 2 mg intravenously. In two studies, psilocybin was administered either in combination with spiritual practice support (Griffiths et al., 2018) or to experienced meditators (Smigielski et al., 2019b). Compared with our analysis, both studies reported a similar pattern of responses for visual and auditory alterations for the administered doses, but substantially larger effect sizes for *Oceanic Boundlessness* and smaller effect sizes for *Dread of Ego Dissolution* than predicted by our dose–response estimates.

### Comparison with effects of other psychedelics

Other classic psychedelics may induce comparable subjective experiences, considering that participants in early studies failed to discriminate between the subjective experiences of psilocybin, lysergic acid diethylamide (LSD) and mescaline (Hollister and Hartman, 1962; Wolbach et al., 1962). So far, no dose–response relationships have been established for those substances, but a report by Liechti (2017) analysed data on LSD from three studies performed by their research group, which showed a comparable pattern of response on the 11-ASC and MEQ30. Whereas the scales *Audio-Visual Synesthesia* and *Changed Meaning of Percepts* exhibited a strong dose–response for LSD, these scales were barely dose-dependent in our analysis. The *Mystical* scale on the MEQ30 was also less dose-dependent and showed smaller effect sizes compared with our results. Similarly, studies with DMT and 5-methoxy-N,N-dimethyltryptamine (5-MeO-DMT) report a largely comparable pattern of responses on both versions of the ASC rating scale (Uthaug et al., 2020), MEQ30 (Barsuglia et al., 2018) and HRS (Gouzoulis-Mayfrank et al., 2005; Strassman et al., 1994). Nevertheless, the comparisons are limited by the small amount of available data, so the characterization of differences requires the utilization of standardized questionnaires in future research to establish dose–response profiles, which could then serve as a general reference to compare and infer subjective experiences.

### Limitations

The results of the present study need to be understood in relation to the method-immanent limitations. First and most generally, the study of subjective experiences depends on introspection and is more challenging to assess than other physiological parameters, especially because these experiences often go beyond the previously experienced epistemic range (Cardeña and Pekala, 2014; Majić et al., 2015). Second, dose–response relationships are, like most biological processes, typically sigmoidal relationships. Since the available data did not cover the upper and lower bounds to resemble a sigmoid function, we used a linear model to approximate the active range of the sigmoid. Consequently, the obtained models are not suited to predict responses for very high and very low doses (e.g. microdosing). Third, while the RVE permits the inclusion of statistically dependent effect sizes (due to repeated measurements) to obtain reliable meta-regression estimates, it is not intended to provide precise variance parameter estimates, nor test null hypotheses regarding heterogeneity parameters (Tanner-Smith et al., 2016). Finally, the generalization of our results is limited by the number of available studies in which the inclusion of participants was highly selective and prone to self-selection bias. Therefore, the obtained results do not necessarily apply to the general population or recreational use aside from controlled laboratory experiments.

## Conclusion

In conclusion, psilocybin intensified almost all characteristics of ASC that were measured by the given questionnaires. The subjective experience induced by psilocybin was mainly characterized by perceptual alterations and positively experienced ego dissolution with the ability to occasion mystical-type experiences. Even though the subjective psilocybin experience is also determined by non-pharmacological variables, we established robust dose–response relationships for most factors and scales, which may be used as a general reference for relating expected and observed dose-specific effects. The results do not necessarily generalize to recreational use, as our analyses were based on data from controlled laboratory experiments in healthy, highly selected study participants. Future research should facilitate comparison of subjective experiences by utilizing standardized questionnaires to improve dose–response profiles and inform future clinical studies.

## Supplemental Material

sj-pdf-1-jop-10.1177_0269881121992676 – Supplemental material for Dose–response relationships of psilocybin-induced subjective experiences in humans Click here for additional data file. Supplemental material, sj-pdf-1-jop-10.1177_0269881121992676 for Dose–response relationships of psilocybin-induced subjective experiences in humans by Tim Hirschfeld and Timo T Schmidt in Journal of Psychopharmacology

## References

[bibr1-0269881121992676] Agin-Liebes GI Malone T Yalch MM, et al. (2020) Long-term follow-up of psilocybin-assisted psychotherapy for psychiatric and existential distress in patients with life-threatening cancer. Journal of Psychopharmacology 34: 155–166. 31916890 10.1177/0269881119897615

[bibr2-0269881121992676] Barrett FS Griffiths RR (2017) The factor structure of the Mystical Experience Questionnaire (MEQ): Reply to Bouso et al., 2016. Human Psychopharmacology 32: 2564. 10.1002/hup.2564 28120488

[bibr3-0269881121992676] Barrett FS Johnson MW Griffiths RR (2015) Validation of the revised Mystical Experience Questionnaire in experimental sessions with psilocybin. Journal of Psychopharmacology 29: 1182–1190. 26442957 10.1177/0269881115609019 PMC5203697

[bibr4-0269881121992676] Barrett FS Johnson MW Griffiths RR (2017) Neuroticism is associated with challenging experiences with psilocybin mushrooms. Personality and Individual Differences 117: 155–160. 28781400 10.1016/j.paid.2017.06.004 PMC5540159

[bibr5-0269881121992676] Barsuglia J Davis AK Palmer R, et al. (2018) Intensity of mystical experiences occasioned by 5-MeO-DMT and comparison with a prior psilocybin study. Frontiers in Psychology 9. 30574112 10.3389/fpsyg.2018.02459 PMC6292276

[bibr6-0269881121992676] Bayne T Hohwy J Owen AM (2016) Are there levels of consciousness? Trends in Cognitive Sciences 20: 405–413. 27101880 10.1016/j.tics.2016.03.009

[bibr7-0269881121992676] Bernasconi F Schmidt A Pokorny T, et al. (2014) Spatiotemporal brain dynamics of emotional face processing modulations induced by the serotonin 1A/2A receptor agonist psilocybin. Cerebral Cortex 24: 3221–3231. 23861318 10.1093/cercor/bht178

[bibr8-0269881121992676] Bogenschutz MP Forcehimes AA Pommy JA, et al. (2015) Psilocybin-assisted treatment for alcohol dependence: A proof-of-concept study. Journal of Psychopharmacology 29: 289–299. 25586396 10.1177/0269881114565144

[bibr9-0269881121992676] Borenstein M Hedges L Rothstein H (2007) Meta-analysis: Fixed effect vs. random effects. Meta-analysis. com. 10.1002/jrsm.12 26061376

[bibr10-0269881121992676] Borenstein M Higgins JPT Hedges LV, et al. (2017) Basics of meta-analysis: I2 is not an absolute measure of heterogeneity. Research Synthesis Methods 8: 5–18. 28058794 10.1002/jrsm.1230

[bibr11-0269881121992676] Brown RT Nicholas CR Cozzi NV, et al. (2017) Pharmacokinetics of escalating doses of oral psilocybin in healthy adults. Clinical Pharmacokinetics 56: 1543–1554. 28353056 10.1007/s40262-017-0540-6

[bibr12-0269881121992676] Carbonaro TM Johnson MW Hurwitz E, et al. (2018) Double-blind comparison of the two hallucinogens psilocybin and dextromethorphan: Similarities and differences in subjective experiences. Psychopharmacology 235: 521–534. 29116367 10.1007/s00213-017-4769-4 PMC6645364

[bibr13-0269881121992676] Cardeña E Pekala RJ (2014) Researching states of consciousness and anomalous experiences. In: Cardeña E Lynn SJ Krippner S (eds) Varieties of Anomalous Experience: Examining the Scientific Evidence, 2nd edn. Washington: American Psychological Association, pp.21–56.

[bibr14-0269881121992676] Cardeña EE Lynn SJE Krippner SE (2000) Varieties of Anomalous Experience: Examining the Scientific Evidence. Washington, DC: American Psychological Association.

[bibr15-0269881121992676] Carhart-Harris RL Williams TM Sessa B, et al. (2011) The administration of psilocybin to healthy, hallucinogen-experienced volunteers in a mock-functional magnetic resonance imaging environment: A preliminary investigation of tolerability. Journal of Psychopharmacology 25: 1562–1567. 20395317 10.1177/0269881110367445

[bibr16-0269881121992676] Carhart-Harris RL Bolstridge M Rucker J, et al. (2016) Psilocybin with psychological support for treatment-resistant depression: An open-label feasibility study. The Lancet Psychiatry 3: 619–627. 27210031 10.1016/S2215-0366(16)30065-7

[bibr17-0269881121992676] Carhart-Harris RL Bolstridge M Day CMJ, et al. (2018) Psilocybin with psychological support for treatment-resistant depression: Six-month follow-up. Psychopharmacology 235: 399–408. 29119217 10.1007/s00213-017-4771-x PMC5813086

[bibr18-0269881121992676] Carter OL Pettigrew JD Hasler F, et al. (2005) Modulating the rate and rhythmicity of perceptual rivalry alternations with the mixed 5-HT 2A and 5-HT 1A agonist psilocybin. Neuropsychopharmacology 30: 1154–1162. 15688092 10.1038/sj.npp.1300621

[bibr19-0269881121992676] Carter OL Burr DC Pettigrew JD, et al. (2005) Using psilocybin to investigate the relationship between attention, working memory, and the serotonin 1A and 2A receptors. Journal of Cognitive Neuroscience 17: 1497–1508. 16269092 10.1162/089892905774597191

[bibr20-0269881121992676] Carter OL Hasler F Pettigrew JD, et al. (2007) Psilocybin links binocular rivalry switch rate to attention and subjective arousal levels in humans. Psychopharmacology 195: 415–424. 17874073 10.1007/s00213-007-0930-9

[bibr21-0269881121992676] Davis AK Barrett FS May DG, et al. (2020) Effects of psilocybin-assisted therapy on major depressive disorder. JAMA Psychiatry. Epub ahead of print 4 November 2020. DOI: 10.1001/jamapsychiatry.2020.3285. PMC7643046 33146667

[bibr22-0269881121992676] Deeks JJ Higgins J Altman DG, et al. (2011) Chapter 9: Analysing data and undertaking meta-analyses. In: Higgins JPT Green S (eds) Cochrane Handbook for Systematic Reviews of Interventions Version 5.1. 0 (updated March 2011). The Cochrane Collaboration, p.2.

[bibr23-0269881121992676] Dittrich A (1994) 50 Years of LSD: Current Status and Perspective of Hallucinogens. New York: Parthenon Publishing, pp.27–42.

[bibr24-0269881121992676] Dittrich A Lamparter D Maurer M (2006) 5D-ABZ: Fragebogen zur Erfassung Aussergewöhnlicher Bewusstseinszustände. Eine kurze Einführung [5D-ASC: Questionnaire for the assessment of altered states of consciousness. A short introduction]. Zürich, Switzerland.

[bibr25-0269881121992676] Dittrich A Lamparter D Maurer M (2010) 5D-ASC: Questionnaire for the Assessment of Altered States of Consciousness. A Short Introduction, 3rd edn. Zürich, Switzerland: PSIN PLUS.

[bibr26-0269881121992676] Fischman MW Johanson CE (1998) Ethical and practical issues involved in behavioral pharmacology research that administers drugs of abuse to human volunteers. Behavioural Pharmacology 9: 479–498. 9862072 10.1097/00008877-199811000-00002

[bibr27-0269881121992676] Fisher Z Tipton E (2015) robumeta: An R-package for robust variance estimation in meta-analysis. arXiv preprint arXiv:1503.02220.

[bibr28-0269881121992676] Garcia-Romeu A Griffiths RR Johnson MW (2015) Psilocybin-occasioned mystical experiences in the treatment of tobacco addiction. Current Drug Abuse Reviews 7: 157–164. 10.2174/1874473708666150107121331 PMC4342293 25563443

[bibr29-0269881121992676] Gouzoulis-Mayfrank E Heekeren K Neukirch A, et al. (2005) Psychological effects of (S)-ketamine and N,N-dimethyltryptamine (DMT): A double-blind, cross-over study in healthy volunteers. Pharmacopsychiatry 38: 301–311. 16342002 10.1055/s-2005-916185

[bibr30-0269881121992676] Griffiths RR Richards WA McCann U, et al. (2006) Psilocybin can occasion mystical-type experiences having substantial and sustained personal meaning and spiritual significance. Psychopharmacology 187: 268–283. 16826400 10.1007/s00213-006-0457-5

[bibr31-0269881121992676] Griffiths RR Richards WA Johnson MW, et al. (2008) Mystical-type experiences occasioned by psilocybin mediate the attribution of personal meaning and spiritual significance 14 months later. Journal of psychopharmacology 22: 621–632. 18593735 10.1177/0269881108094300 PMC3050654

[bibr32-0269881121992676] Griffiths RR Johnson MW Richards WA, et al. (2011) Psilocybin occasioned mystical-type experiences: Immediate and persisting dose-related effects. Psychopharmacology 218: 649–665. 21674151 10.1007/s00213-011-2358-5 PMC3308357

[bibr33-0269881121992676] Griffiths RR Johnson MW Carducci MA, et al. (2016) Psilocybin produces substantial and sustained decreases in depression and anxiety in patients with life-threatening cancer: A randomized double-blind trial. Journal of Psychopharmacology 30: 1181–1197. 27909165 10.1177/0269881116675513 PMC5367557

[bibr34-0269881121992676] Griffiths RR Johnson MW Richards WA, et al. (2018) Psilocybin-occasioned mystical-type experience in combination with meditation and other spiritual practices produces enduring positive changes in psychological functioning and in trait measures of prosocial attitudes and behaviors. Journal of Psychopharmacology 32: 49–69. 29020861 10.1177/0269881117731279 PMC5772431

[bibr35-0269881121992676] Grob CS Danforth AL Chopra GS, et al. (2011) Pilot study of psilocybin treatment for anxiety in patients with advanced-stage cancer. Archives of General Psychiatry 68: 71–78. 20819978 10.1001/archgenpsychiatry.2010.116

[bibr36-0269881121992676] Haijen EC Kaelen M Roseman L, et al. (2018) Predicting responses to psychedelics: A prospective study. Frontiers in Pharmacology 9: 897. 30450045 10.3389/fphar.2018.00897 PMC6225734

[bibr37-0269881121992676] Hartogsohn I (2017) Constructing drug effects: A history of set and setting. Drug Science, Policy and Law 3: 205032451668332.

[bibr38-0269881121992676] Hasler F Grimberg U Benz MA, et al. (2004) Acute psychological and physiological effects of psilocybin in healthy humans: A double-blind, placebo-controlled dose–effect study. Psychopharmacology 172: 145–156. 14615876 10.1007/s00213-003-1640-6

[bibr39-0269881121992676] Hedges LV Tipton E Johnson MC (2010) Robust variance estimation in meta-regression with dependent effect size estimates. Research Synthesis Methods 1: 39–65. 26056092 10.1002/jrsm.5

[bibr40-0269881121992676] Hempel S Miles JN Booth MJ, et al. (2013) Risk of bias: A simulation study of power to detect study-level moderator effects in meta-analysis. Systematic Reviews 2: 107. 24286208 10.1186/2046-4053-2-107 PMC4219184

[bibr41-0269881121992676] Hemsley DR Ward ES (1985) Individual differences in reaction to the abuse of LSD. Personality and Individual Differences 6: 515–517.

[bibr42-0269881121992676] Higgins JPT Thompson SG (2002) Quantifying heterogeneity in a meta-analysis. Statistics in Medicine 21: 1539–1558. 12111919 10.1002/sim.1186

[bibr43-0269881121992676] Hofmann A Heim R Brack A, et al. (1958) Psilocybin, ein psychotroper Wirkstoff aus dem mexikanischen RauschpilzPsilocybe mexicana Heim. Experientia 14: 107–109. 13537892 10.1007/BF02159243

[bibr44-0269881121992676] Hollister LE Hartman AM (1962) Mescaline, lysergic acid diethylamide and psilocybin: Comparison of clinical syndromes, effects on color perception and biochemical measures. Comprehensive Psychiatry 3: 235–241. 13908449 10.1016/s0010-440x(62)80024-8

[bibr45-0269881121992676] Hyde RW (1960) Psychological and social determinants of drug action. In: Sarwer-Foner GJ (ed.) The Dynamics of Psychiatric Drug Therapy, 1st edn. Springfield, IL: Thomas, pp.297–315.

[bibr46-0269881121992676] James W (1906) The Varieties of Religious Experience. A Study in Human Nature.(1902).

[bibr47-0269881121992676] Jenkins DG Quintana-Ascencio PF (2020) A solution to minimum sample size for regressions. PLoS One 15: e0229345. 32084211 10.1371/journal.pone.0229345 PMC7034864

[bibr48-0269881121992676] Johnson MW Richards WA Griffiths RR (2008) Human hallucinogen research: Guidelines for safety. Journal of psychopharmacology (Oxford, England) 22: 603–620. 10.1177/0269881108093587 PMC3056407 18593734

[bibr49-0269881121992676] Johnson MW Garcia-Romeu A Cosimano MP, et al. (2014) Pilot study of the 5-HT2AR agonist psilocybin in the treatment of tobacco addiction. Journal of Psychopharmacology 28: 983–992. 25213996 10.1177/0269881114548296 PMC4286320

[bibr50-0269881121992676] Johnson MW Garcia-Romeu A Griffiths RR (2017) Long-term follow-up of psilocybin-facilitated smoking cessation. The American Journal of Drug and Alcohol Abuse 43: 55–60. 27441452 10.3109/00952990.2016.1170135 PMC5641975

[bibr51-0269881121992676] Kometer M Schmidt A Bachmann R, et al. (2012) Psilocybin biases facial recognition, goal-directed behavior, and mood state toward positive relative to negative emotions through different serotonergic subreceptors. Biological Psychiatry 72: 898–906. 22578254 10.1016/j.biopsych.2012.04.005

[bibr52-0269881121992676] Leary T Litwin GH Metzner R (1963) Reactions to psilocybin administered in a supportive environment. Journal of Nervous and Mental Disease 137: 561–573. 10.1097/00005053-196312000-00007 14087676

[bibr53-0269881121992676] Lewis CR Preller KH Braden BB, et al. (2020) Rostral anterior cingulate thickness predicts the emotional psilocybin experience. Biomedicines 8: 34. 10.3390/biomedicines8020034 PMC7168190 32085521

[bibr54-0269881121992676] Liechti ME (2017) Modern clinical research on LSD. Neuropsychopharmacology 42: 2114–2127. 28447622 10.1038/npp.2017.86 PMC5603820

[bibr55-0269881121992676] Lindenblatt H Krämer E Holzmann-Erens P, et al. (1998) Quantitation of psilocin in human plasma by high-performance liquid chromatography and electrochemical detection: Comparison of liquid–liquid extraction with automated on-line solid-phase extraction. Journal of Chromatography B: Biomedical Sciences and Applications 709: 255–263. 9657222 10.1016/s0378-4347(98)00067-x

[bibr56-0269881121992676] MacLean KA Johnson MW Griffiths RR (2011) Mystical experiences occasioned by the hallucinogen psilocybin lead to increases in the personality domain of openness. Journal of Psychopharmacology 25: 1453–1461. 21956378 10.1177/0269881111420188 PMC3537171

[bibr57-0269881121992676] MacLean KA Leoutsakos J-MS Johnson MW, et al. (2012) Factor analysis of the mystical experience questionnaire: A study of experiences occasioned by the hallucinogen psilocybin. Journal for the Scientific Study of Religion 51: 721–737. 23316089 10.1111/j.1468-5906.2012.01685.x PMC3539773

[bibr58-0269881121992676] Madsen MK Fisher PM Stenbæk DS, et al. (2020) A single psilocybin dose is associated with long-term increased mindfulness, preceded by a proportional change in neocortical 5-HT2A receptor binding. European Neuropsychopharmacology 33: 71–80. 32146028 10.1016/j.euroneuro.2020.02.001

[bibr59-0269881121992676] Majić T Schmidt TT Gallinat J (2015) Peak experiences and the afterglow phenomenon: When and how do therapeutic effects of hallucinogens depend on psychedelic experiences? Journal of Psychopharmacology 29: 241–253. 25670401 10.1177/0269881114568040

[bibr60-0269881121992676] Metzner R Litwin G Weil G (1965) The relation of expectation and mood to psilocybin reactions: A questionnaire study. Psychedelic Review 5: 3–39.

[bibr61-0269881121992676] Nakazawa M (2019) Fmsb: Functions for Medical Statistics Book with Some Demographic Data. R package version 0.5 2. Available at: https://CRAN.R-project.org/package=fmsb (accessed 25 February 2020).

[bibr62-0269881121992676] Nicholas CR Henriquez KM Gassman MC, et al. (2018) High dose psilocybin is associated with positive subjective effects in healthy volunteers. Journal of Psychopharmacology 32: 770–778. 29945469 10.1177/0269881118780713 PMC7751062

[bibr63-0269881121992676] Nichols DE (2016) Psychedelics. Pharmacological Reviews 68: 264–355. 26841800 10.1124/pr.115.011478 PMC4813425

[bibr64-0269881121992676] Nutt DJ King LA Nichols DE (2013) Effects of schedule I drug laws on neuroscience research and treatment innovation. Nature Reviews Neuroscience 14: 577–585. 10.1038/nrn3530 23756634

[bibr65-0269881121992676] Pahnke WN (1963) Drugs and mysticism: An analysis of the relationship between psychedelic drugs and the mystical consciousness. Unpublished Doctoral Dissertation, Harvard University Press.

[bibr66-0269881121992676] Pahnke WN (1966) Drugs and mysticism. International Journal of Parapsychology 8: 295–314.

[bibr67-0269881121992676] Passie T (2007) Bewusstseinszustände: Konzeptualisierung Und Messung. LIT Verlag Münster.

[bibr68-0269881121992676] Pokorny T Preller KH Kraehenmann R, et al. (2016) Modulatory effect of the 5-HT1A agonist buspirone and the mixed non-hallucinogenic 5-HT1A/2A agonist ergotamine on psilocybin-induced psychedelic experience. European Neuropsychopharmacology 26: 756–766. 26875114 10.1016/j.euroneuro.2016.01.005

[bibr69-0269881121992676] Pokorny T Preller KH Kometer M, et al. (2017) Effect of psilocybin on empathy and moral decision-making. International Journal of Neuropsychopharmacology 20: 747–757. 10.1093/ijnp/pyx047 PMC5581487 28637246

[bibr70-0269881121992676] Preller KH Pokorny T Hock A, et al. (2016) Effects of serotonin 2A/1A receptor stimulation on social exclusion processing. Proceedings of the National Academy of Sciences 113: 5119–5124. 10.1073/pnas.1524187113 PMC4983864 27091970

[bibr71-0269881121992676] Quednow BB Kometer M Geyer MA, et al. (2012) Psilocybin-induced deficits in automatic and controlled inhibition are attenuated by ketanserin in healthy human volunteers. Neuropsychopharmacology 37: 630–640. 21956447 10.1038/npp.2011.228 PMC3260978

[bibr72-0269881121992676] R Core Team (2019) R: A Language and Environment for Statistical Computing. R Foundation for Statistical Computing, Vienna, Austria. Available at: http://www.r-project.org/index.html.

[bibr73-0269881121992676] Riba J Rodríguez-Fornells A Strassman RJ, et al. (2001) Psychometric assessment of the Hallucinogen Rating Scale. Drug and Alcohol Dependence 62: 215–223. 11295326 10.1016/s0376-8716(00)00175-7

[bibr74-0269881121992676] Roseman L Nutt DJ Carhart-Harris RL (2018) Quality of acute psychedelic experience predicts therapeutic efficacy of psilocybin for treatment-resistant depression. Frontiers in Pharmacology 8: 974. 29387009 10.3389/fphar.2017.00974 PMC5776504

[bibr75-0269881121992676] Ross S Bossis A Guss J, et al. (2016) Rapid and sustained symptom reduction following psilocybin treatment for anxiety and depression in patients with life-threatening cancer: A randomized controlled trial. Journal of Psychopharmacology 30: 1165–1180. 27909164 10.1177/0269881116675512 PMC5367551

[bibr76-0269881121992676] Russ SL Carhart-Harris RL Maruyama G, et al. (2019a) Replication and extension of a model predicting response to psilocybin. Psychopharmacology 236: 3221–3230. 31203401 10.1007/s00213-019-05279-z

[bibr77-0269881121992676] Russ SL Carhart-Harris RL Maruyama G, et al. (2019b) States and traits related to the quality and consequences of psychedelic experiences. Psychology of Consciousness: Theory, Research, and Practice 6: 1–21.

[bibr78-0269881121992676] Schmidt A Bachmann R Kometer M, et al. (2012) Mismatch negativity encoding of prediction errors predicts S-ketamine-induced cognitive impairments. Neuropsychopharmacology 37: 865–875. 22030715 10.1038/npp.2011.261 PMC3280661

[bibr79-0269881121992676] Schmidt A Kometer M Bachmann R, et al. (2013) The NMDA antagonist ketamine and the 5-HT agonist psilocybin produce dissociable effects on structural encoding of emotional face expressions. Psychopharmacology 225: 227–239. 22836372 10.1007/s00213-012-2811-0

[bibr80-0269881121992676] Schmidt T Majic T (2016) Empirische Untersuchung veränderter Bewusstseinszustände., pp.1–25.

[bibr81-0269881121992676] Schmidt TT (2017) The Altered States Database (ASDB). Available at: https://osf.io/8mbru/ (accessed 25 February 2020).

[bibr82-0269881121992676] Schmidt TT Berkemeyer H (2018) The altered states database: Psychometric data of altered states of consciousness. Frontiers in Psychology 9: 1028. 30013493 10.3389/fpsyg.2018.01028 PMC6036510

[bibr83-0269881121992676] Schultes RE Hofmann A (1979) Plants of the Gods: Origins of Hallucinogenic Use. New York: McGraw-Hill.

[bibr84-0269881121992676] Smigielski L Kometer M Scheidegger M, et al. (2019a) Characterization and prediction of acute and sustained response to psychedelic psilocybin in a mindfulness group retreat. Scientific Reports 9: 1–13. 31649304 10.1038/s41598-019-50612-3 PMC6813317

[bibr85-0269881121992676] Smigielski L Scheidegger M Kometer M, et al. (2019b) Psilocybin-assisted mindfulness training modulates self-consciousness and brain default mode network connectivity with lasting effects. NeuroImage 196: 207–215. 30965131 10.1016/j.neuroimage.2019.04.009

[bibr86-0269881121992676] Smigielski L Kometer M Scheidegger M, et al. (2020) P300-mediated modulations in self–other processing under psychedelic psilocybin are related to connectedness and changed meaning: A window into the self–other overlap. Human Brain Mapping 41: 4982–4996. 32820851 10.1002/hbm.25174 PMC7643385

[bibr87-0269881121992676] Stace WT (1960) Mysticism and Philosophy. Philadelphia: Lippincott.

[bibr88-0269881121992676] Strassman RJ Qualls CR Uhlenhuth EH, et al. (1994) Dose-response study of N,N-dimethyltryptamine in humans. II. Subjective effects and preliminary results of a new rating scale. Archives of General Psychiatry 51: 98–108. 8297217 10.1001/archpsyc.1994.03950020022002

[bibr89-0269881121992676] Studerus E Gamma A Vollenweider FX (2010) Psychometric evaluation of the altered states of consciousness rating scale (OAV). PLoS One 5: e12412. 20824211 10.1371/journal.pone.0012412 PMC2930851

[bibr90-0269881121992676] Studerus E Kometer M Hasler F, et al. (2011) Acute, subacute and long-term subjective effects of psilocybin in healthy humans: A pooled analysis of experimental studies. Journal of Psychopharmacology 25: 1434–1452. 20855349 10.1177/0269881110382466

[bibr91-0269881121992676] Studerus E Gamma A Kometer M, et al. (2012) Prediction of psilocybin response in healthy volunteers. PLoS One 7: e30800. 22363492 10.1371/journal.pone.0030800 PMC3281871

[bibr92-0269881121992676] Tanner-Smith EE Tipton E (2014) Robust variance estimation with dependent effect sizes: practical considerations including a software tutorial in Stata and SPSS. Research Synthesis Methods 5: 13–30. 26054023 10.1002/jrsm.1091

[bibr93-0269881121992676] Tanner-Smith EE Tipton E Polanin JR (2016) Handling complex meta-analytic data structures using robust variance estimates: A tutorial in R. Journal of Developmental and Life-Course Criminology 2: 85–112.

[bibr94-0269881121992676] Tipton E (2015) Small sample adjustments for robust variance estimation with meta-regression. Psychological Methods 20: 375. 24773356 10.1037/met0000011

[bibr95-0269881121992676] Tylš F Páleníček T Horáček J (2014) Psilocybin–summary of knowledge and new perspectives. European Neuropsychopharmacology 24: 342–356. 24444771 10.1016/j.euroneuro.2013.12.006

[bibr96-0269881121992676] Umbricht D Koller R Vollenweider FX, et al. (2002) Mismatch negativity predicts psychotic experiences induced by NMDA receptor antagonist in healthy volunteers. Biological Psychiatry 51: 400–406. 11904134 10.1016/s0006-3223(01)01242-2

[bibr97-0269881121992676] Uthaug MV Lancelotta R Szabo A, et al. (2020) Prospective examination of synthetic 5-methoxy-N,N-dimethyltryptamine inhalation: Effects on salivary IL-6, cortisol levels, affect, and non-judgment. Psychopharmacology 237: 773–785. 31822925 10.1007/s00213-019-05414-w PMC7036074

[bibr98-0269881121992676] Vollenweider FX Kometer M (2010) The neurobiology of psychedelic drugs: Implications for the treatment of mood disorders. Nature Reviews Neuroscience 11: 642–651. 20717121 10.1038/nrn2884

[bibr99-0269881121992676] Vollenweider FX Preller KH (2020) Psychedelic drugs: Neurobiology and potential for treatment of psychiatric disorders. Nature Reviews Neuroscience 21: 611–624. 32929261 10.1038/s41583-020-0367-2

[bibr100-0269881121992676] Vollenweider FX Vollenweider-Scherpenhuyzen MFI Bäbler A, et al. (1998) Psilocybin induces schizophrenia-like psychosis in humans via a serotonin-2 agonist action. NeuroReport 9: 3897–3902. 9875725 10.1097/00001756-199812010-00024

[bibr101-0269881121992676] Vollenweider FX Csomor PA Knappe B, et al. (2007) The effects of the preferential 5-HT2A agonist psilocybin on prepulse inhibition of startle in healthy human volunteers depend on interstimulus interval. Neuropsychopharmacology 32: 1876–1887. 17299516 10.1038/sj.npp.1301324

[bibr102-0269881121992676] Wittmann M Carter O Hasler F, et al. (2007) Effects of psilocybin on time perception and temporal control of behaviour in humans. Journal of Psychopharmacology 21: 50–64. 16714323 10.1177/0269881106065859

[bibr103-0269881121992676] Wolbach AB Miner EJ Isbell H (1962) Comparison of psilocin with psilocybin, mescaline and LSD-25. Psychopharmacologia 3: 219–223. 14007905 10.1007/BF00412109

[bibr104-0269881121992676] Zinberg NE (1984) Drug, Set, and Setting: The Basis for Controlled Intoxicant Use. New Haven: Yale University Press.

