# Supplementary material for: Dose–response relationships of psilocybin-induced subjective experiences in humans
Source: J Psychopharmacol. 2021 Mar 4;35(4):384–97. doi: 10.1177/0269881121992676 (PMC8058832; doi:10.1177/0269881121992676)
Supplement: sj-pdf-1-jop-10.1177_0269881121992676 – Supplemental material for Dose–response relationships of psilocybin-induced subjective experiences in humans [file sj-pdf-1-jop-10.1177_0269881121992676.pdf]

Supplement

Supplementary material for:

Title: Dose-response relationships of psilocybin-induced subjective experiences in humans

Authors: Hirschfeld T, Schmidt TT

Results for the additional meta-regression analysis including patient data

The additionally included data comprised data on patients with alcohol use disorder (Bogenschutz et al., 2015), treatment-resistant major depression (Carhart-Harris et al., 2018), major depressive disorder (Davis et al., 2020) and three studies on patients with cancer-related psychiatric distress (Grob et al., 2011; Griffiths et al., 2016; Ross et al., 2016), as specified in **Table S1**.

Study	Sample Size	Study description	Data report	Psilocybin administration
Grob et al., 2011	N=12	Pilot study for treatment of anxiety for patients with life-threatening cancer, double-blind, placebo controlled	5D-ASC	Oral administration as gelatin capsules<br>Dosage:<br>(1) 200 µg/kg body weight
Bogenschutz et al., 2015	N=10/6	Patients with alcohol addiction with additional Motivational Enhancement Therapy, single-group proof-of-concept study with two sessions	5D-ASC	Oral administration as gelatin capsules<br>Dosage:<br>(1) 300 µg/kg body weight (N=10)<br>(2) 400 µg/kg body weight (N=6)
Griffiths et al., 2016	(1): N=24<br>(2): N=25	Patients with psychological distress (depression, anxiety) due to life-threatening cancer, randomized, double-blind cross-over design including a placebo condition	5D-ASC<br>HRS<br>MEQ30	Oral administration as gelatin capsules<br>Dosages:<br>(1) 14 or 43 µg/kg body weight (1 or 3 mg/70kg)<br>(2) 314 or 429 µg/kg body weight (22 or 30mg/70kg)
Ross et al., 2016	(1): N=14<br>(2): N=12	Patients with psychological distress (depression, anxiety) due to life-threatening cancer, randomized, double-blind cross-over design including a placebo condition	MEQ30	Oral administration as gelatin capsules<br>Dosage:<br>(1/2) 300 µg/kg body weight
Carhart-Harris et al., 2018 (Subsample: Carhart-Harris et al., 2016)	N=20<br>(N=12)	Patients with (mostly) severe, unipolar, treatment-resistant major depression, open-label trial in supporting setting	11-ASC	Oral administration as gelatin capsules<br>Dosages:<br>(1) 143 µg/kg body weight (10 mg)<br>(2) 357 µg/kg body weight (25mg)
Davis et al., 2020	N=24	Patients with major depressive disorder, randomized controlled trial with delayed treatment group, single-blind, two sessions	MEQ30	Oral administration as gelatin capsules<br>Dosages:<br>(1) 286 µg/kg body weight (20 mg/70kg)<br>(2) 429 µg/kg body weight (30 mg/70kg)

Table S1: Summary of studies that were additionally included in the meta-regression analysis comprising patient data. Studies can contain multiple samples (e.g. from the application of different dosages). The following amount of patient data was complemented to the main analysis: 5 observations (extracted from 3 studies) for the 5D-ASC; 2 observations (extracted from 1 study) for the 11-ASC; 6 observation (extracted from 3 studies) for the MEQ30; 2 observation (extracted from 1 study) for the HRS.

Together with the data from healthy participants this results in: 19 observations (extracted from 10 studies) for the 5D-ASC; 12 observations (extracted from 8 studies) for the 11-ASC; 17 observation (extracted from 7 studies) for the MEQ30; 10 observation (extracted from 4 studies) for the HRS.

Outcome	Intercept Coeff.	Intercept (95 % CI)	Intercept SE	Slope Coeff.	Slope (95 % CI)	Slope SE	t (df)	p	Tau²	I²
5D-ASC										
Auditory Alterations	3.7	(-7.4 − 14.9)	2.33	0.031	(0.003 − 0.061)	0.0061	5.0 (1.7)	.049	0.6	5.0
Oceanic Boundlessness	16.3	(-6.9 – 39.4)	7.92	0.087	(-0.010 − 0.183)	0.0330	2.6 (3.6)	.066	117.7	76.6
Dread of Ego Dissolution	2.4	(-10.4 – 15.3)	3.79	0.059	(0.005 − 0.113)	0.0161	3.7 (2.7)	.040	16.7	50.1
Vigilance Reduction	19.3	(3.9 − 34.6)	4.75	0.039	(-0.035 − 0.112)	0.0208	1.9 (2.6)	.177	49.1	60.8
Visionary Restructuralization	14.7	(-4.8 – 34.3)	6.67	0.111	(0.030 − 0.193)	0.0277	4.0 (3.5)	.021	71.5	68.1
11-ASC										
Anxiety	-2.0	(-9.1 − 5.1)	1.68	0.042	(-0.016 − 0.100)	0.0125	3.4 (1.9)	.086	14.0	74.1
Audio Visual Synesthesia	19.1	(-23.5 – 61.7)	12.36	0.081	(-0.169 − 0.332)	0.0593	1.4 (2.0)	.301	263.4	83.7
Blissful State	11.7	(-11.9 – 35.4)	7.14	0.129	(-0.010 − 0.249)	0.0312	4.2 (2.3)	.042	13.5	32.0
Complex Imagery	20.9	(-21.2 – 62.9)	11.62	0.118	(-0.091 − 0.327)	0.0507	2.3 (2.1)	.140	50.8	54.3
Changed Meaning of Percepts	28.2	(6.0 – 50.3)	6.39	0.018	(-0.185 − 0.220)	0.0459	0.4 (2.0)	.738	116.6	79.1
Disembodiment	10.3	(-28.5 − 49.1)	10.89	0.088	(-0.164 − 0.341)	0.0599	1.5 (2.0)	.276	93.6	71.2
Elementary Imagery	29.9	(-27.7 – 87.6)	16.16	0.100	(-0.200 − 0.400)	0.0707	1.4 (2.0)	.292	118.2	71.8
Experience of Unity	6.6	(-4.8 − 18.1)	2.91	0.112	(0.040 − 0.185)	0.0191	5.9 (2.3)	.020	0.0	0.0
Insightfulness	7.0	(-10.6 – 24.5)	5.34	0.112	(0.002 − 0.221)	0.0279	4.0 (2.2)	.048	24.7	42.9
Impaired Control & Cognition	16.7	(5.1 – 28.3)	3.17	0.010	(-0.097 − 0.117)	0.0239	0.4 (1.9)	.717	30.1	65.0
Spiritual Experience	-12.5	(-24.7 − -0.4)	3.65	0.157	(0.055 − 0.259)	0.0269	5.8 (2.3)	.020	71.7	80.0
MEQ30										
Ineffability	40.1	(7.2 – 72.9)	9.05	0.098	(0.026 − 0.170)	0.0244	4.0 (3.5)	.021	45.3	61.1
Mystical	26.9	(4.5 – 49.2)	5.30	0.099	(0.049 − 0.150)	0.0163	6.1 (3.2)	.008	15.7	28.7
Positive Mood	42.5	(16.8 – 68.2)	6.87	0.077	(0.012 − 0.142)	0.0217	3.6 (3.4)	.031	36.4	56.2
Transcendence of Time & Space	26.2	(3.2 – 49.3)	5.86	0.106	(0.054 − 0.159)	0.0174	6.1 (3.3)	.007	33.5	53.9
HRS										
Affect	1.00	(0.03 − 1.97)	0.17	0.003	(0.001 − 0.004)	0.0004	6.7 (2.3)	.015	0.01	48.0
Cognition	0.76	(-0.16 − 1.68)	0.17	0.004	(0.002 − 0.006)	0.0005	7.3 (2.4)	.011	0.04	69.9
Intensity	1.64	(0.49 − 2.80)	0.24	0.003	(0.001 − 0.005)	0.0007	4.5 (2.6)	.028	0.03	74.2
Perception	0.53	(-0.71 − 1.76)	0.17	0.004	(0.002 − 0.005)	0.0004	9.0 (2.0)	.011	0.01	27.3
Somaesthesia	0.76	(-0.53 − 2.04)	0.25	0.003	(0.000 − 0.005)	0.0006	4.3 (2.5)	.032	0.05	83.0
Volition	1.26	(0.56 − 1.97)	0.12	0.001	(0.000 − 0.002)	0.0002	5.3 (2.3)	.025	0.01	53.7

Table S2: Meta-regression estimates of the additional analysis for all included questionnaires with respective factors/dimensions/subscales. Coefficients (Coeff.) are presented with 95 % confidence intervals (CI) and standard errors (SE). The t-test statistic determines if a linear relationship exists under the null hypothesis that the slope is equal to zero. Tau^2 indicates the between-study variance and I^2 indicates the degree of inconsistency across studies in percent. Intercepts' estimates are rounded to the first decimal, except for the HRS due to its different range (0-4). Slope estimates are rounded to the third decimal considering its greater sensitivity to increasing dose.

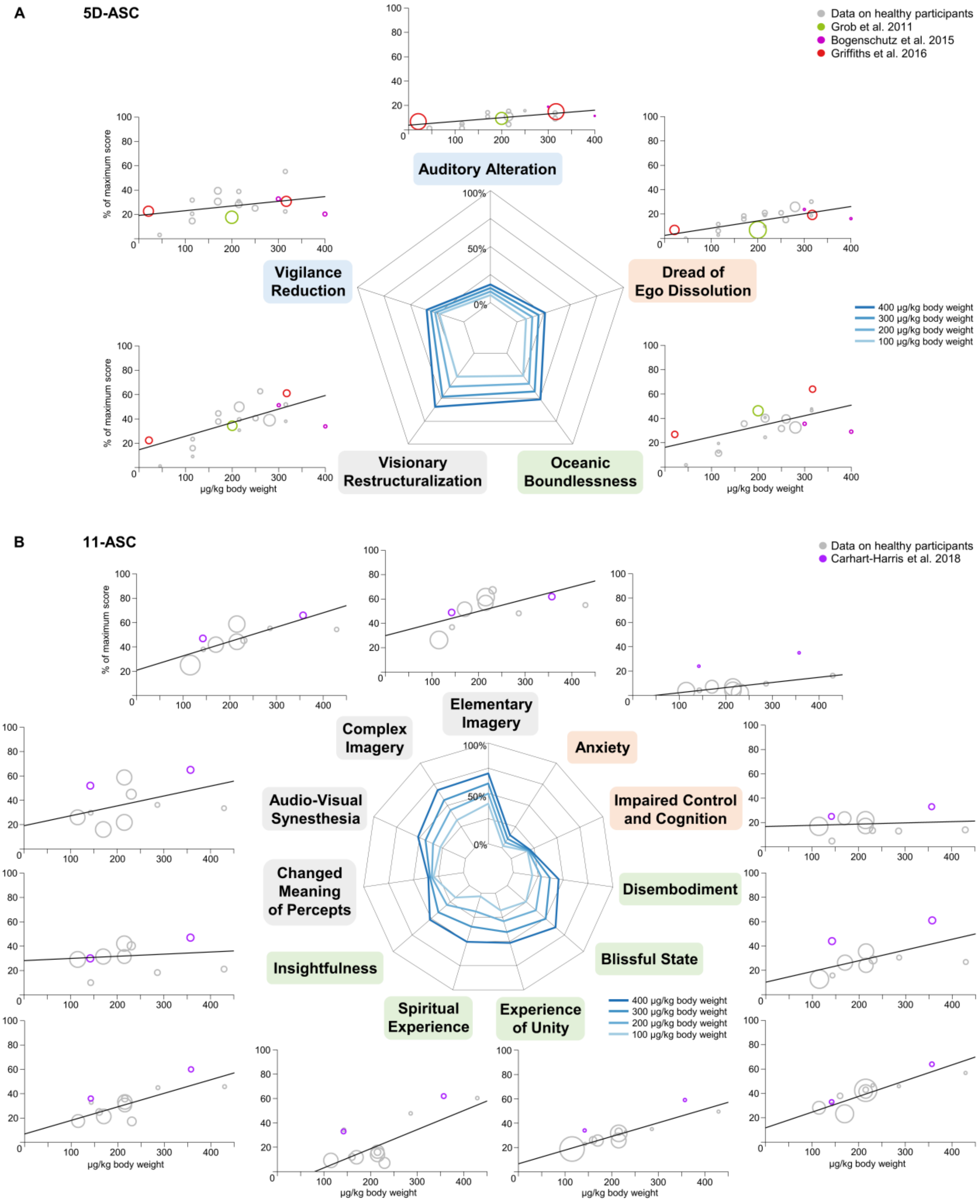

Figure S1: Dose-response relationships for the Altered States of Consciousness Rating Scale including patient data

Dose-specific subjective effects of psilocybin in patients and healthy study participants measured with the Altered States of Consciousness Rating scale. The data of this instrument can be analyzed according to a schema where items are organized into five factors, called "dimensions" of ASC experiences (5D-ASC) (see **A**). A finer-grained quantification of specific aspects of subjective experiences is obtained when the questionnaire is analyzed according to an eleven factors schema (**B**). These eleven factors can be considered as subscales of the three core dimensions of the 5D-ASC, namely "Oceanic Boundlessness", "Dread of Ego Dissolution" and "Visionary Restructuralization" (see corresponding colouring of the subscale names). Doses are given as µg per kg body weight; effects are given as percentage scored of the maximum score on each factor. The colour of the circles indicates data from the same sample of participants (same colour corresponds to dependent data), while the circle size represents the weight of the data based on study variance (see Methods). Spiderplots present the estimated dose-responses for 100 - 400 µg/kg body weight, corresponding to the range of doses which were included in the respective analysis. As compared to the main analysis, which was comprised of data from healthy participants only, here we included patient data from Grob et al. (2011), Bogenschutz et al. (2015) and Griffiths et al. (2016) on the 5D-ASC and Carhart-Harris et al. (2018) on the 11-ASC.

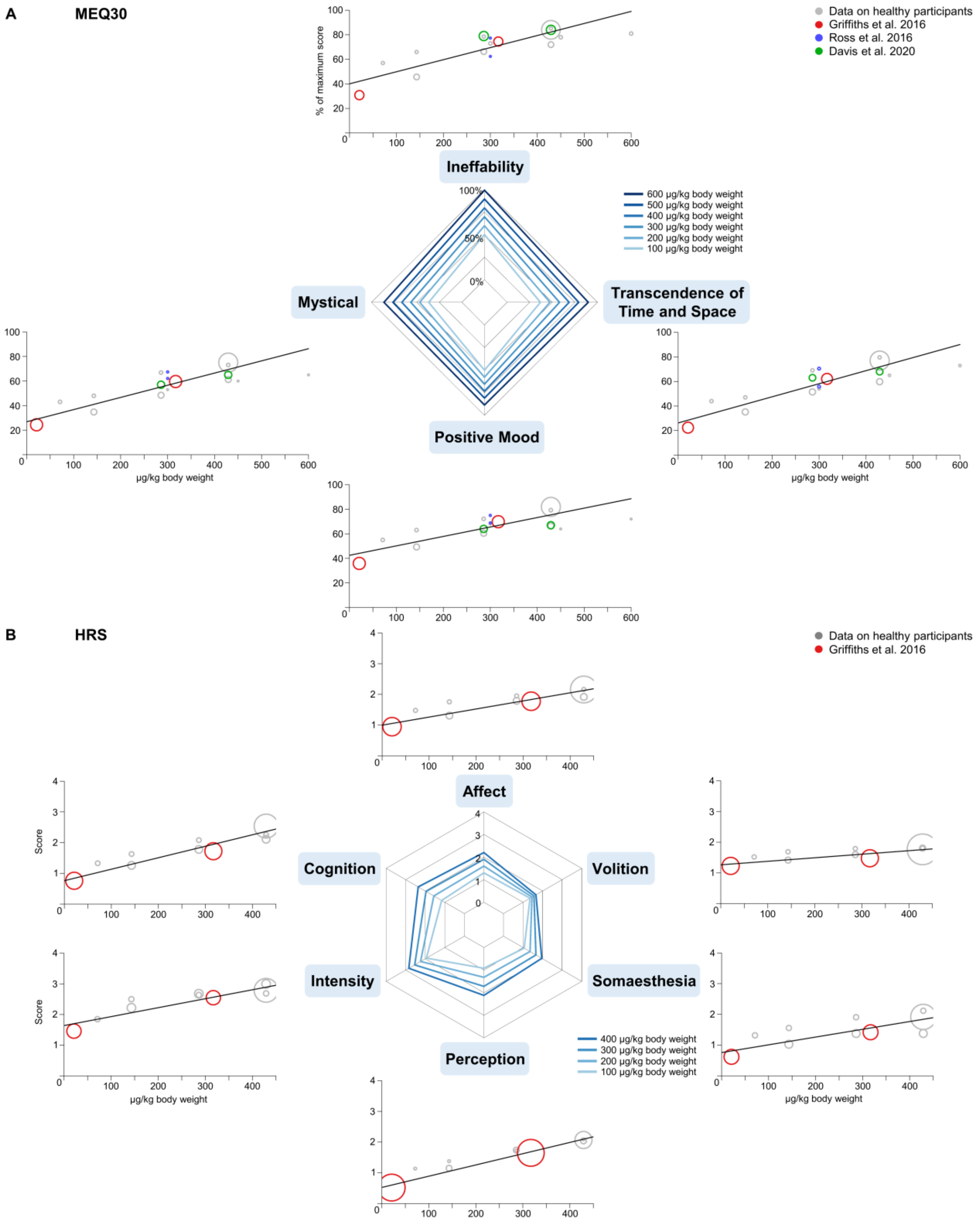

Figure S2: Dose-response relationships for MEQ30 and HRS including patient data

Dose-specific subjective effects of psilocybin for the psychometric instruments MEQ30 (**A**) and HRS (**B**). Doses are given as µg per kg body weight. Effects on the MEQ30 are presented as percentage scored on the maximum score. Effects on the HRS range from 0 – 4 (items in the questionnaire from 0 ”not at all” to 4 “extreme”). The colour of the circles indicates data from the same sample of participants (same colour corresponds to dependent data), the circle size represents the weight of the data based on study variance (see Methods). Spiderplots present the estimated dose-responses for 100 - 600 µg/kg body weight on the MEQ30 and 100 - 400 µg/kg body weight on the HRS, corresponding to the range of doses which were included in the respective analysis. As compared to the main analysis, which was comprised of data from healthy participants only, here we included patient data from Griffiths et al. (2016), Ross et al. (2016) and Davis et al. (2020) on the MEQ30 and Griffiths et al. (2016) on the HRS.

References

Bogenschutz MP, Forcehimes AA, Pommy JA, et al. (2015) Psilocybin-assisted treatment for alcohol dependence: a proof-of-concept study. *Journal of psychopharmacology* 29(3): 289–299.

Carhart-Harris RL, Bolstridge M, Rucker J, et al. (2016) Psilocybin with psychological support for treatment-resistant depression: an open-label feasibility study. *The Lancet Psychiatry* 3(7). Elsevier: 619–627. DOI: 10.1016/S2215-0366(16)30065-7.

Carhart-Harris RL, Bolstridge M, Day CMJ, et al. (2018) Psilocybin with psychological support for treatment-resistant depression: six-month follow-up. *Psychopharmacology* 235(2): 399–408. DOI: 10.1007/s00213-017-4771-x.

Davis AK, Barrett FS, May DG, et al. (2020) Effects of Psilocybin-Assisted Therapy on Major Depressive Disorder. *JAMA Psychiatry*. DOI: 10.1001/jamapsychiatry.2020.3285.

Griffiths RR, Johnson MW, Carducci MA, et al. (2016) Psilocybin produces substantial and sustained decreases in depression and anxiety in patients with life-threatening cancer: A randomized double-blind trial. *Journal of psychopharmacology* 30(12): 1181–1197.

Grob CS, Danforth AL, Chopra GS, et al. (2011) Pilot study of psilocybin treatment for anxiety in patients with advanced-stage cancer. *Archives of general psychiatry* 68(1). American Medical Association: 71–78.

Ross S, Bossis A, Guss J, et al. (2016) Rapid and sustained symptom reduction following psilocybin treatment for anxiety and depression in patients with life-threatening cancer: a randomized controlled trial. *Journal of psychopharmacology* 30(12): 1165–1180.
